# CTLA-4 Genetic Variants Predict Survival in Patients with Sepsis

**DOI:** 10.3390/jcm8010070

**Published:** 2019-01-10

**Authors:** Caspar Mewes, Benedikt Büttner, José Hinz, Ayelet Alpert, Aron-Frederik Popov, Michael Ghadimi, Tim Beissbarth, Mladen Tzvetkov, Ole Jensen, Julius Runzheimer, Michael Quintel, Shai Shen-Orr, Ingo Bergmann, Ashham Mansur

**Affiliations:** 1 Department of Anesthesiology, University Medical Center, Georg August University, D-37075 Goettingen, Germany; caspar.mewes@med.uni-goettingen.de (C.M.); benedikt.buettner@med.uni-goettingen.de (B.B.); julius.runzheimer@med.uni-goettingen.de (J.R.); mquintel@med.uni-goettingen.de (M.Q.); ingo.bergmann@med.uni-goettingen.de (I.B.); 2 Department of Anesthesiology and Intensive Care Medicine, Klinikum Region Hannover, D-30459 Hannover, Germany; jose.hinz@krh.eu; 3 Faculty of Medicine, Technion–Israeli Institute of Technology, 31096 Haifa, Israel; ayelethappy@gmail.com (A.A.); shenorr@technion.ac.il (S.S.-O.); 4 Department of Thoracic and Cardiovascular Surgery, University Medical Center, Eberhard Karls University, D-72076 Tuebingen, Germany; aronf.popov@gmail.com; 5 Department of General and Visceral Surgery, University Medical Center, Georg August University, D-37075 Goettingen, Germany; mghadim@med.uni-goettingen.de; 6 Department of Medical Bioinformatics, University Medical Center, Georg August University, D-37077 Goettingen, Germany; tim.beissbarth@ams.med.uni-goettingen.de; 7 Department of Pharmacology, University Medical Center, Ernst-Moritz-Arndt-University, D-17487 Greifswald, Germany; mladen.tzvetkov@uni-greifswald.de; 8 Department of Clinical Pharmacology, University Medical Center, Georg August University, D-37075 Goettingen, Germany; ole.jensen@stud.uni-goettingen.de

**Keywords:** sepsis, CTLA-4, single nucleotide polymorphisms, haplotypes, survival, predictors

## Abstract

Cytotoxic T lymphocyte-associated protein 4 (CTLA-4) is a coinhibitory checkpoint protein expressed on the surface of T cells. A recent study by our working group revealed that the rs231775 single nucleotide polymorphism (SNP) in the CTLA-4 gene was associated with the survival of patients with sepsis and served as an independent prognostic variable. To further investigate the impact of CTLA-4 genetic variants on sepsis survival, we examined the effect of two functional SNPs, CTLA-4 rs733618 and CTLA-4 rs3087243, and inferred haplotypes, on the survival of 644 prospectively enrolled septic patients. Kaplan–Meier survival analysis revealed significantly lower 90-day mortality for rs3087243 G allele carriers (*n* = 502) than for AA-homozygous (*n* = 142) patients (27.3% vs. 40.8%, *p* = 0.0024). Likewise, lower 90-day mortality was observed for TAA haplotype-negative patients (*n* = 197; compound rs733618 T/rs231775 A/rs3087243 A) than for patients carrying the TAA haplotype (*n* = 447; 24.4% vs. 32.9%, *p* = 0.0265). Carrying the rs3087243 G allele hazard ratio (HR): 0.667; 95% confidence interval (CI): 0.489–0.909; *p* = 0.0103) or not carrying the TAA haplotype (HR: 0.685; 95% CI: 0.491–0.956; *p* = 0.0262) remained significant covariates for 90-day survival in the multivariate Cox regression analysis and thus served as independent prognostic variables. In conclusion, our findings underscore the significance of CTLA-4 genetic variants as predictors of survival of patients with sepsis.

## 1. Introduction

Sepsis is defined as life-threatening organ dysfunction caused by a dysregulated host response to infection [1] and remains a leading cause of death and critical illness worldwide [2]. The reported incidence of sepsis is increasing in aging populations with more comorbidities [1,3,4] and is accompanied by rising health care costs. The multifaceted pathophysiology of sepsis and sepsis-related organ dysfunction is highly diverse and therefore has been intensively investigated in the recent past [5]. Host genetic characteristics, such as gene variants encoding innate immune effectors or inflammatory mediators, seem to contribute considerably to the disease course and sepsis outcome [6,7,8,9]. The genetic component of host susceptibility to sepsis is clearly polygenetic [10], and identification of responsible variants will help elucidate the pathophysiology and biology of the disease. Identifying sepsis-associated genetic variants and evaluating their impacts on gene expression, protein function and host physiology will potentially reveal future targets for sepsis treatment [11].

CTLA-4 has gained recognition since James P. Allison and Tasuku Honja were awarded the Nobel Prize for Physiology or Medicine in 2018 for their discovery of cancer therapy by inhibition of negative immune regulation [12]. CTLA-4 is a coinhibitory cell surface protein expressed on T cells that competes with CD28 for binding to CD80 and CD86 on antigen-presenting cells (APCs) [13,14] and thereby reduces T cell activation and proliferation [15]. Thus, an increased CTLA-4 expression has an inhibitory effect on the host immune reaction [16].

Coinhibitory immune checkpoint proteins, such as PD-1 and CTLA-4, have been reported to play key roles in the course of sepsis [13,17,18,19], and our working group has revealed genetic associations of their genes with the survival of septic patients [9,20]. Our recent study revealed that the CTLA-4 rs231775 GG genotype, which was reported to have an attenuated inhibitory effect on T cell reactions [21] and was associated with greater T cell activation and proliferation and autoimmune diseases [22], was also associated with favorable 90- and 28-day survival in septic patients compared to the survival rates of A allele carriers at this position [20].

The CTLA-4 gene is located on chromosome 2q33 and contains two further functional polymorphisms, the CTLA-4 rs733618 and CTLA-4 rs3087243, which are associated with enhanced T cell reactions and higher susceptibility to autoimmune diseases, such as myasthenia gravis [23], systemic lupus erythematosus [24,25], Hashimoto thyroiditis [26], latent autoimmune diabetes [27], multiple sclerosis [28] and acute liver transplant rejection [29]. This study aimed to investigate the impact of these two functional single nucleotide polymorphisms (SNPs) and inferred haplotypes compound of rs733618, rs231775, and rs3087243 on the 90-day mortality of septic patients in order to further reveal the role of CTLA-4 genetic variants in sepsis.

## 2. Methods

### 2.1. Patients

The present study included 644 prospectively enrolled adult Caucasian patients with sepsis. Patients were recruited from three surgical intensive care units (ICUs) through the GENOSEP database of the Department of Anesthesiology at the University Medical Center Goettingen [20]. Enrolled patients were identified by daily screening of the three ICUs according to the actual sepsis definitions and guidelines [1,30]. The exclusion criteria were defined in previous studies [20,31] and were an age below 18 years, pregnancy, therapy with immunosuppressive drugs or chemotherapy, myocardial infarction within six weeks before enrollment, human immunodeficiency virus (HIV) infection, chronic heart failure classified as New York Heart Association (NYHA) stage IV, end-stage incurable disease, a persistent vegetative state (apallic syndrome), and a “Do Not Treat” (DNT) or “Do Not Resuscitate” (DNR) order. Upon enrollment, blood was drawn within 72 h, and the patients were followed up for 90 days unless previously dismissed or deceased.

This investigation and the experimental protocols were approved under ethical project identification code 15/1/12 by the institutional ethics committee of the University of Goettingen in Goettingen, Germany. The study was performed in accordance with the provisions of the Declaration of Helsinki and relevant guidelines and regulations. The methods were carried out in accordance with approved guidelines. Written informed consent was obtained from either the patient or their legal representative.

### 2.2. Data Collection

All patient data were recorded through the GENOSEP database. At enrollment, relevant baseline characteristics were obtained, such as comorbidities, preexisting medication, and the initial Sequential Organ Failure Assessment (SOFA) and Acute Physiology and Chronic Health Evaluation (APACHE II) scores. Patients were followed up for 90 days, and 90-day mortality was recorded as the primary outcome parameter. Within the first 28 days after sepsis onset, relevant clinical data were generated through a standardized clinical report form (CRF) on a daily basis. The data were collected from the electronic patient record system (Intellispace Critical Care and Anesthesia (ICCA), Phillips Healthcare, Andover, MA, USA). Parameters included SOFA score-relevant organ dysfunction variables; information about organ support requirements; and inflammatory, kidney and liver values.

### 2.3. CTLA-4 Genotyping and Haplotyping

DNA extraction, CTLA-4 rs733618 and rs3087243 genotyping, and haplotyping were performed entirely in the laboratories of the Department of Clinical Pharmacology of the University Medical Center Goettingen. The DNA extraction and genotyping methods were performed as previously described [20]. DNA was automatically extracted from either 200 µL of ethylene diamine tetraacetic acid (EDTA) blood using the QIAamp^®^ DNA Blood Kit in the QIAcube^®^, 350 µL of EDTA blood using the EZ1^®^ DNA Blood Kit in the BioRobot EZ1^®^, or peripheral blood mononuclear cells (PBMCs) using the AllPrep DNA Mini Kit according to the manufacturer’s instructions (all from Qiagen, Hilden, Germany). The quantity and quality of the DNA were determined by spectrophotometric measurement. Genotyping of the extracted DNA was performed via TaqMan polymerase chain reaction (PCR) using the predesigned TaqMan^®^ SNP Genotyping Assays C_2415791_10 and C_3296043_10 and the 7900HT Fast Real-Time PCR System (Applied Biosystems, Foster City, CA, USA) according to the manufacturer’s instructions (Life Technologies, Darmstadt, Germany). The genotyping outcomes were generated using the 7900HT Fast Real-Time PCR System Software (SDS v2.4.1 for Windows 7, Applied Biosystems, Foster City, CA, USA). Twenty percent of the samples were genotyped in duplicate for reliability assessment and yielded concordant results.

The CTLA-4 haplotypes were inferred using the PHASE software (Matthew Stephens, Washington, DC, USA, version 2.1), which is a Bayesian statistical method that is used to reconstruct haplotypes from population genotype data [32,33]. CTLA-4 haplotype reconstruction was performed in 100 iterations to yield the most likely haplotype combination (best pair) for each individual, with a minimum probability for the most likely combination of 99.9%.

### 2.4. Statistical Analyses

The data analysis was performed using the STATISTICA 13 software (version 13.0, StatSoft, Tulsa, OK, USA). The patient baseline characteristics and disease severity data were evaluated in univariate analyses with Pearson’s Chi-square test, a two-sided Fisher’s exact test, the Mann–Whitney U test or the Kruskal-Wallis test, if applicable. The Kaplan–Meier time-to-event data were compared with the log-rank test. Multivariate Cox regression analyses were used to adjust for possible effect confounders. The appropriateness of the genotype distributions and allele frequencies of the study population was evaluated with a Chi-square test of the Hardy–Weinberg equilibrium. The CTLA-4 rs733618 CC and CT genotypes, as well as the rs3087243 GG and AG genotypes, were pooled to compare the clinical course of the functionally relevant rs733618 homozygous TT (*n* = 550) with C allele carriers (*n* = 94) and the rs3087243 G allele carriers (*n* = 502) with homozygous AA (*n* = 142) patients.

A *p*-value <0.05 was considered statistically significant. Data are presented as absolute numbers or percentages for categorical variables and the mean ± standard deviation for continuous variables.

### 2.5. Data Availability

The datasets generated and/or analyzed during the current study are available from the corresponding author on reasonable request.

## 3. Results

### 3.1. Baseline Characteristics

Our cohort of 644 prospectively enrolled adult Caucasian patients with sepsis, which was previously described [20], was examined in the present study. The average age was 63 ± 15 years; 66% were of male gender; and the average SOFA and APACHE II scores were 9.4 ± 3.9 and 22 ± 7, respectively, upon enrollment. A total of 93% of the patients were mechanically ventilated, 79% received vasopressor therapy, 21% received renal replacement therapy, and 51% were in septic shock during the observation period (Table 1 and Table 2).

**Figure 1:**
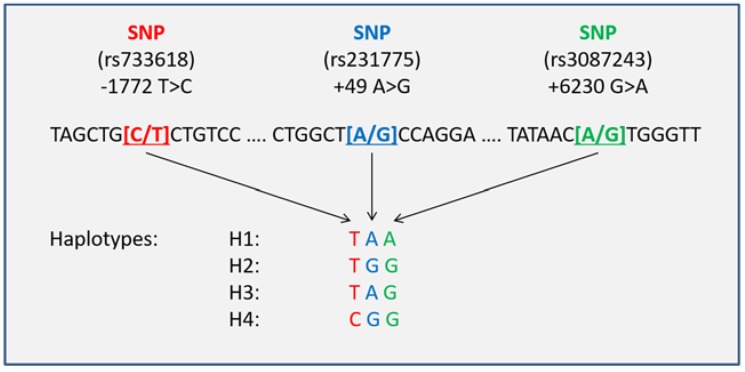
Compound cytotoxic T lymphocyte-associated protein 4 (CTLA-4) haplotypes. SNP, single nucleotide polymorphism.

All patients were successfully genotyped, and the relevant haplotypes were inferred. The CTLA-4 rs733618 and rs3087243 genotype distributions were 4:90:550 (CC:CT:TT) and 142:305:197 (AA:AG:GG), with observed minor allele frequencies (MAFs) of 0.08 and 0.47, respectively. The observed frequencies were consistent with the Hardy–Weinberg equilibrium (*χ*^2^ test *p* = 0.8789 and *p* = 0.2448) and almost equaled the reported MAFs from reference populations, such as HapMap CEPH (CEU) with MAFs of 0.06 and 0.46 respectively (dbSNP database, accession numbers ss79540 [34] and ss44315036 [35]). The CTLA-4 haplotype analysis compound of the rs733618, rs231775, and rs3087243 SNPs revealed four common haplotypes (H1: TAA, H2: TGG, H3: TAG, and H4: CGG) (Figure 1).

The corresponding haplotype distributions can be obtained from Table 3. Linkage disequilibrium (LD) analysis of the three SNPs revealed a squared correlation coefficient (*r*^2^) of 0.39 for rs231775 and rs3087243, 0.23 for rs733618 and rs231775, and 0.09 for rs733618 and rs3087243.

Analysis of the baseline characteristics with regard to the genotype distributions revealed a significant difference in age between rs3087243 G allele carriers (*n* = 502) and AA homozygous patients (*n* = 142) at sepsis onset (62 ± 15 vs. 67 ± 14 years; *p* = 0.0033; Table 1). In addition, noncarriers of the inferred haplotype H1: TAA (TAA-negative, *n* = 197) received significantly more renal replacement therapy upon sepsis onset (15% vs. 6%, *p* = 0.0005; Table 2) as well as over the observation period (26% vs. 18%; *p* = 0.0204; Table 2) than carriers of the H1: TAA haplotype (TAA-positive, *n* = 447). These significant differences in patient baseline characteristics were adjusted by their inclusion as potential confounders in the Cox regression analysis.

### 3.2. Outcomes

**Figure 2:**
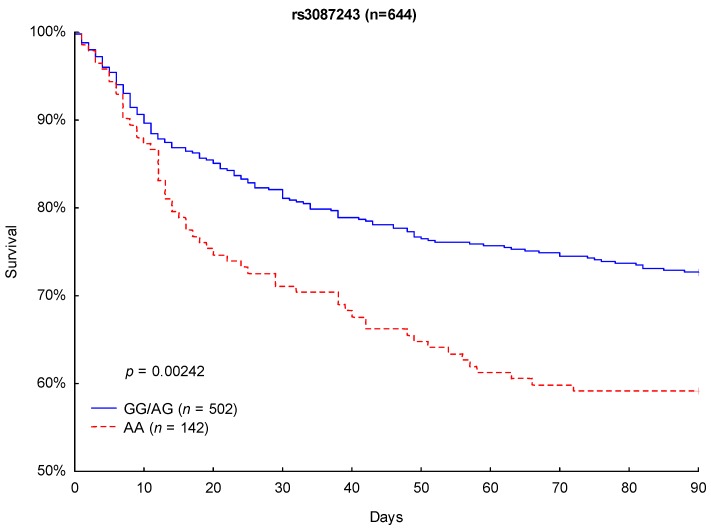
Kaplan–Meier survival analysis (90 days) with regard to the CTLA-4 rs3087243 genotypes.

**Figure 3:**
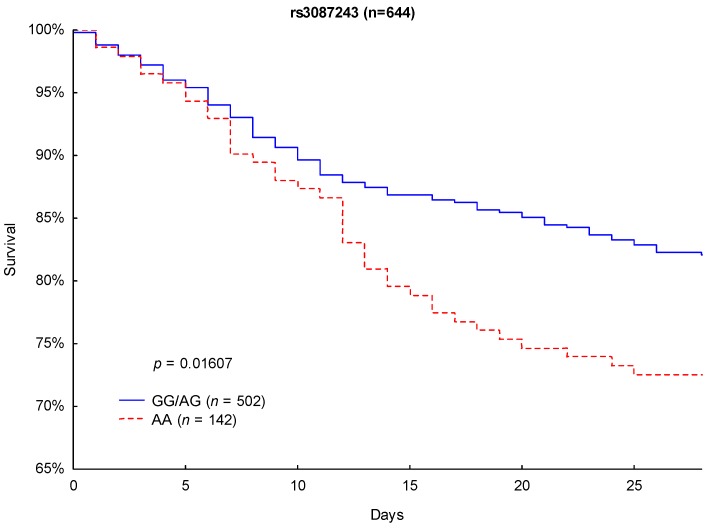
Kaplan–Meier survival analysis (28 days) with regard to the CTLA-4 rs3087243 genotypes.

Although Kaplan–Meier analysis of 90- and 28-day survival revealed no significant differences between the CTLA-4 rs733618 TT homozygous and C allele carriers (*p* = 0.6864 and *p* = 0.4693), significant differences in survival were observed between the CTLA-4 rs3087243 G allele carriers and the AA-homozygous patients. CTLA-4 rs3087243 G allele carriers showed significantly lower 90-day mortality (27.3% vs. 40.8%, respectively; *p* = 0.0024; Figure 2) and 28-day mortality (17.9% vs. 27.5%; *p* = 0.0161; Figure 3) than AA homozygous patients.

**Figure 4:**
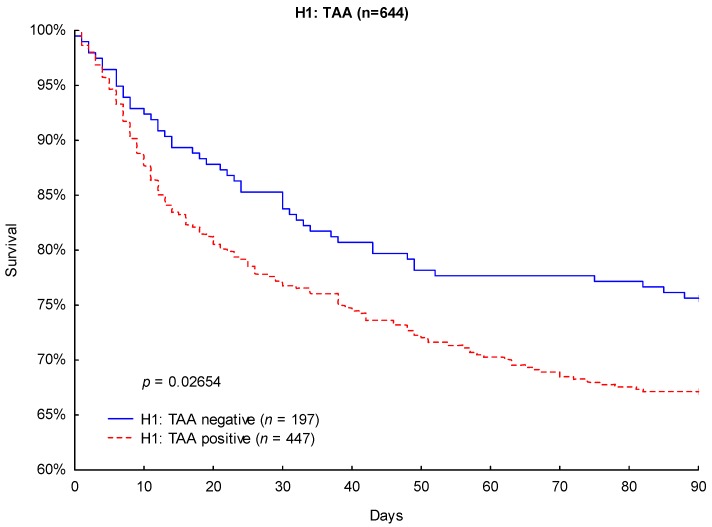
Kaplan–Meier survival analysis (90 days) with regard to the CTLA-4 haplotype H1: TAA.

**Figure 5:**
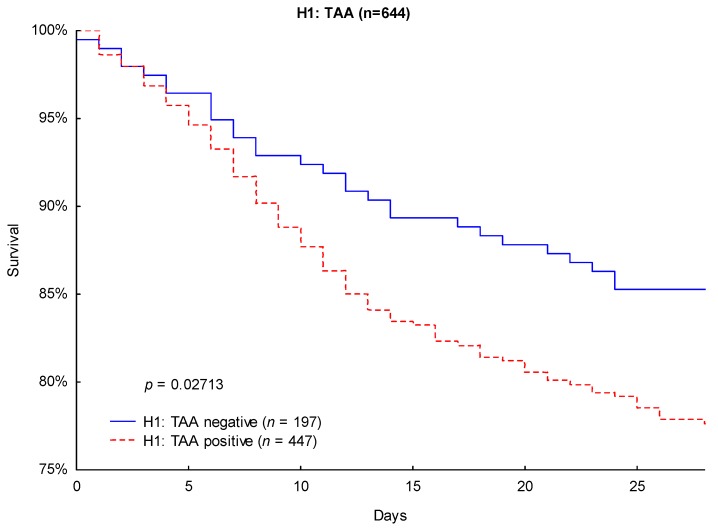
Kaplan–Meier survival analysis (28 days) with regard to the CTLA-4 haplotype H1: TAA.

Likewise, Kaplan–Meier survival analysis revealed significantly lower 90-day mortality (24.4% vs. 32.9%; *p* = 0.0265; Figure 4) and 28-day mortality (14.7% vs. 22.4%; *p* = 0.0271; Figure 5) in TAA-negative patients compared to that of patients carrying the H1: TAA haplotype.

No other significant differences in mortality were observed for the remaining inferred haplotypes (H2: TGG, H3: TAG, and H4: CGG).

### 3.3. Cox Regression Analysis

Univariate and multivariate Cox regression analyses with regard to the CTLA-4 rs3087243 genotypes and the H1: TAA haplotype were performed to adjust for possible effects of baseline characteristics and other potential confounders on 90- and 28-day mortality. In addition to the baseline variables age, male gender, body mass index (BMI), and SOFA and APACHE II scores, statin therapy was included in accordance with findings from our previous study [36]. Application of renal replacement therapy upon sepsis onset and during the observation period was included in the Cox regression analysis for the H1: TAA haplotype based on our findings in the patient baseline characteristics. Multivariate Cox regression analyses revealed that carrying the CTLA-4 rs3087243 G allele was a significant positive predictor for 90-day survival (hazard ratio: 0.667; 95% confidence interval (CI): 0.489–0.909; *p* = 0.0103; Table 4) and 28-day survival (hazard ratio: 0.676; 95% CI: 0.463–0.987; *p* = 0.0423; Table 4), as was not carrying the H1:TAA haplotype (for 90-day survival, hazard ratio: 0.685; 95% CI: 0.491–0.956; *p* = 0.0262; for 28-day survival, hazard ratio: 0.621; 95% CI: 0.407–0.947; *p* = 0.0270; Table 5).

### 3.4. Disease Severity

The average SOFA and organ-specific SOFA subscores, as well as inflammatory, kidney, and liver parameters were included in the disease severity analysis (Table 6 and Table 7). Assessment of disease severity revealed that CTLA-4 rs3087243 G allele carriers had significantly lower average SOFA-Central Nervous System (SOFA-CNS) scores (1.9 ± 1.1 vs. 2.2 ± 1.0; *p* = 0.0094) over the observation period and a lower percentage of ventilated days as a fraction of observation days (65 ± 32% vs. 71 ± 32%; *p* = 0.0280) than AA homozygous patients.

The H1: TAA noncarriers showed highly significant higher average procalcitonin levels (5.1 ± 9.7 ng/dL vs. 3.5 ± 9.1 ng/dL; *p* = 0.0002) as well as significantly lower urine outputs (2792 ± 1242 mL/day vs. 3060 ± 1370 mL/day; *p* = 0.0229) over the observation period than the H1: TAA-positive patients.

## 4. Discussion

Identification of genetic variants associated with the outcome of sepsis is important for understanding the pathophysiology of the disease and identifying patients at a higher risk of death, and may even reveal future targets for personalized medicine. The present study aimed to identify associations between functional SNPs and haplotypes of the CTLA-4 gene with the outcomes of septic patients. The main finding was that in addition to our previous identification of the CTLA-4 rs231775 SNP as a prognostic variable for the outcome of septic patients, the functional CTLA-4 rs3087243 SNP and the inferred CTLA-4 haplotype H1: TAA were significantly associated with sepsis survival. Both CTLA-4 rs3087243 G allele carriers and CTLA-4 H1: TAA haplotype-negative patients had significantly better 90- and 28-day survival. To the best of our knowledge, this study is the first to report an association of these genetic variants with sepsis survival. The identified associations remained significant predictors for 90- and 28-day mortality in the multivariate Cox regression analysis, indicating that despite potential confounders, they were independent prognostic variables for the survival of septic patients.

The findings at the CTLA-4 rs3087243 position could be explained by observations that revealed lower CTLA-4 protein expression levels as well as higher susceptibility to autoimmune diseases in rs3087243 G allele carriers [24,26,27,28]. We can assume that lower expression levels of the coinhibitory regulatory CTLA-4 protein in rs3087243 G allele carriers result in an enhanced T lymphocyte host immune response [37], which may be beneficial in the immunosuppressive phase of sepsis. Our hypothesis that genetic variants associated with lower CTLA-4 expression levels and autoimmune disease susceptibility may be favorable during the course of sepsis is further supported by our findings for the H1: TAA haplotype. This most common CTLA-4 haplotype consists of the rs733618 T, rs231775 A, and rs3087243 A alleles, of which the second two are associated with unfavorable sepsis outcomes. Not carrying this haplotype was significantly associated with better survival during the course of sepsis.

Furthermore, our study revealed that CTLA-4 rs3087243 G allele carriers showed significantly better sepsis disease severity in terms of a lower average SOFA-CNS score and a reduced need for organ support, as represented by a reduced number of ventilation days as a fraction of observation days (Table 6). Significantly lower average urine outputs (mL/day) and higher procalcitonin levels were observed for the H1: TAA-negative patients (Table 7). Although the lower urine output levels can be explained by the significantly higher administration of renal replacement therapy to the H1: TAA-negative patients in the patient baseline characteristics, we do not have a satisfactory explanation for the higher average procalcitonin levels.

The present study has some limitations. This study is a single-center study focusing on a relatively large and prospectively enrolled cohort of severely ill patients from surgical ICUs; no patients from medical ICUs were included in the study. Therefore, our observations may not be completely applicable to other ICU cohorts. In addition, our findings should be validated in additional patient cohorts, ideally including patients of other ethnicities and from other ICUs (i.e., neurological or medical ICUs).

## 5. Conclusions 

Although a sepsis-specific biomarker is nonexistent at present, potential for the use of genetic biomarkers for prognostic purposes is high. In this context, our study provides further evidence implicating CTLA-4 polymorphisms and haplotypes in determining sepsis severity and mortality, and thus they may serve as prognostic variables. However, the molecular mechanisms by which CTLA-4 polymorphisms and haplotypes influence the sepsis disease severity and mortality risk remain to be identified. Our findings raise hope that identification of patients at risk and development of tailored therapeutic approaches may be enabled by the effective use of modern molecular diagnostics, including evaluation of genetic variants, such as the SNPs and haplotypes identified in our present study.

## Figures and Tables

**Table 1:** Patient baseline characteristics with regard to the cytotoxic T lymphocyte-associated protein 4 (CTLA-4) rs3087243 genotypes.

Parameter	All (*n* = 644)	rs3087243		*p*-Value
		AA (*n* = 142)	GG/AG (*n* = 502)	
Age (years)	63 ± 15	67 ± 14	62 ± 15	0.0033
Male (%)	66	64	67	0.5560
Body mass index	28 ± 6	27 ± 6	28 ± 6	0.2881
Sepsis severity				
Septic shock (%)	51	52	51	0.8467
SOFA score day 1	9.4 ± 3.9	9.4 ± 3.9	9.4 ± 3.9	0.8047
APACHE II score day 1	22 ± 7	22 ± 7	21 ± 7	0.2844
Comorbidities, *n* (%)				
Hypertension	54	53	55	0.7094
History of myocardial infarction	5	6	5	0.9057
Chronic obstructive pulmonary disease	15	12	16	0.2436
Renal dysfunction	10	8	11	0.3879
Non-insulin-dependent diabetes mellitus	9	8	9	0.8492
Insulin-dependent diabetes mellitus	11	12	10	0.5349
Chronic liver disease	6	6	6	0.9875
History of cancer	16	15	16	0.7830
History of stroke	6	9	5	0.0623
Recent surgical history, *n* (%)				
Elective surgery	29	37	27	
Emergency surgery	53	46	55	
No history of surgery	18	17	18	
Site of infection, *n* (%)				
Lung	62	64	61	
Abdomen	20	20	20	
Bone or soft tissue	4	3	4	
Surgical wound	2	2	1	
Urogenital	2	1	3	
Primary bacteremia	7	6	7	
Other	3	4	4	
Organ support (%)				
Used during observation period				
Mechanical ventilation	93	96	93	0.1850
Use of vasopressor	79	82	78	0.4063
Renal replacement therapy	21	20	21	0.7173
Used on sepsis onset				
Mechanical ventilation	86	86	86	0.9381
Use of vasopressor	67	66	67	0.9048
Renal replacement therapy	9	9	9	0.8851

SOFA, sequential organ failure assessment; APACHE, acute physiology and chronic health evaluation.

**Table 2:** Patient baseline characteristics with regard to the CTLA-4 haplotype H1: TAA.

Parameter	All (*n* = 644)	H1: TAA		*p*-Value
		Positive (*n* = 447)	Negative (*n* = 197)	
Age (years)	63 ± 15	64 ± 15	62 ± 14	0.1014
Male (%)	66	67	64	0.4356
Body mass index	28 ± 6	28 ± 6	28 ± 7	0.2388
Sepsis severity				
Septic shock (%)	51	51	53	0.6384
SOFA score day 1	9.4 ± 3.9	9.3 ± 3.9	9.7 ± 3.8	0.1802
APACHE II score day 1	22 ± 7	21 ± 7	22 ± 7	0.1474
Comorbidities, *n* (%)				
Hypertension	54	54	54	0.9671
History of myocardial infarction	5	6	3	0.0758
Chronic obstructive pulmonary disease	15	15	16	0.5779
Renal dysfunction	10	10	11	0.6734
Non-insulin-dependent diabetes mellitus	9	9	8	0.6654
Insulin-dependent diabetes mellitus	11	9	13	0.1480
Chronic liver disease	6	6	8	0.3893
History of cancer	16	12	17	0.0731
History of stroke	6	6	6	0.8209
Recent surgical history, *n* (%)				
Elective surgery	29	31	26	
Emergency surgery	53	52	54	
No history of surgery	18	17	20	
Site of infection, *n* (%)				
Lung	62	64	58	
Abdomen	20	19	21	
Bone or soft tissue	4	3	6	
Surgical wound	2	2	2	
Urogenital	2	2	3	
Primary bacteremia	7	7	6	
Other	3	3	4	
Organ support (%)				
Used during observation period				
Mechanical ventilation	93	93	93	0.9580
Use of vasopressor	79	79	80	0.6749
Renal replacement therapy	21	18	26	0.0204
Used on sepsis onset				
Mechanical ventilation	86	86	85	0.8341
Use of vasopressor	67	66	69	0.4944
Renal replacement therapy	9	6	15	0.0005

**Table 3:** CTLA-4 haplotype distributions.

Haplotype CTLA-4-1772 T>C/+49 A>G/+6230 G>A	Number of Haplotypes	*n* (%)
H1: TAA	0	197 (30.59)
	1	305 (47.36)
	2	142 (22.05)
H2: TGG	0	316 (49.07)
	1	263 (40.84)
	2	65 (10.09)
H3: TAG	0	451 (70.03)
	1	178 (27.64)
	2	15 (2.33)
H4: CGG	0	550 (85.40)
	1	90 (13.98)
	2	4 (0.62)

**Table 4:** Cox regression analysis with regard to the CTLA-4 rs3087243 genotypes.

**Variable**	**90 Days**					
	**Univariate Analysis**			**Multivariate Analysis**		
	**Hazard Ratio**	**95% CI**	***p*-Value**	**Hazard Ratio**	**95% CI**	***p*-Value**
Age	1.032	1.020–1.043	0.000000	1.025	1.013–1.038	0.000047
Male gender	1.093	0.810–1.474	0.558922	1.076	0.796–1.454	0.633436
Body mass index	0.985	0.961–1.009	0.218173	0.979	0.954–1.005	0.109574
SOFA score day 1	1.104	1.065–1.143	0.000000	1.072	1.025–1.122	0.002432
APACHE II score day 1	1.069	1.046–1.092	0.000000	1.033	1.004–.1062	0.026207
Statin therapy	1.114	0.807–1.536	0.511644	0.979	0.702–1.366	0.901706
CTLA-4 rs3087243 G allele	0.612	0.450–0.833	0.001760	0.667	0.489–0.909	0.010307
	**28 Days**					
	**Univariate Analysis**			**Multivariate Analysis**		
Age	1.029	1.015–1.043	0.000035	1.023	1.009–1.039	0.001824
Male gender	1.278	0.875–1.866	0.204154	1.270	0.868–1.860	0.218759
Body mass index	0.982	0.953–1.013	0.252686	0.976	0.945–1.008	0.135751
SOFA score day 1	1.119	1.071–1.169	0.000000	1.085	1.026–1.146	0.003854
APACHE II score day 1	1.073	1.045–1.102	0.000000	1.033	0.998–1.070	0.063787
Statin therapy	0.964	0.641–1.451	0.861906	0.841	0.552–1.281	0.420505
CTLA-4 rs3087243 G allele	0.624	0.428–0.908	0.013778	0.676	0.463–0.987	0.042323

**Table 5:** Cox regression analysis with regard to the CTLA-4 haplotype H1: TAA.

**Variable**	**90 Days**					
	**Univariate Analysis**			**Multivariate Analysis**		
	**Hazard Ratio**	**95% CI**	***p*-Value**	**Hazard Ratio**	**95% CI**	***p*-Value**
Age	1.032	1.020–1.043	0.000000	1.025	1.013–1.038	0.000049
Male gender	1.093	0.810–1.474	0.558922	1.079	0.798–1.459	0.620112
BMI	0.985	0.961–1.009	0.218173	0.975	0.950–1.001	0.061158
SOFA score day 1	1.104	1.065–1.143	0.000000	1.038	0.988–1.091	0.139057
APACHE II score day 1	1.069	1.046–1.092	0.000000	1.027	0.997–1.057	0.078734
Statin therapy	1.114	0.807–1.536	0.511644	1.086	0.777–1.519	0.629022
Renal replacement therapy during observation period	2.854	2.134–3.817	0.000000	2.724	1.867–3.974	0.000000
Renal replacement therapy upon sepsis onset	1.565	1.021–2.399	0.040064	1.973	1.192–3.268	0.008241
H1: TAA negative	0.698	0.504–0.967	0.030812	0.685	0.491–0.956	0.026202
	**28 Days**					
	**Univariate Analysis**			**Multivariate Analysis**		
Age	1.029	1.015–1.043	0.000035	1.023	1.009–1.039	0.001984
Male gender	1.278	0.875–1.866	0.204154	1.269	0.866–1.859	0.221286
BMI	0.982	0.953–1.013	0.252686	0.972	0.940–1.004	0.089311
SOFA score day 1	1.119	1.071–1.169	0.000000	1.050	0.988–1.116	0.118403
APACHE II score day 1	1.073	1.045–1.102	0.000000	1.026	0.990–1.064	0.155294
Statin therapy	0.964	0.641–1.451	0.861906	0.790	0.517–1.209	0.278234
Renal replacement therapy during observation period	3.028	2.131–4.301	0.000000	2.772	1.769–4.343	0.000009
Renal replacement therapy upon sepsis onset	1.616	0.970–2.691	0.065366	1.933	1.060–3.524	0.031464
H1: TAA negative	0.632	0.418–0.955	0.029340	0.621	0.407–0.947	0.027008

**Table 6:** Disease severity with regard to the CTLA-4 rs3087243 genotypes.

Variable	All (*n* = 644)	rs3087243		*p*-Value
		AA (*n* = 142)	GG/AG (*n* = 502)	
SOFA	7.0 ± 3.5	7.4 ± 3.8	6.9 ± 3.5	0.3005
SOFA-Respiratory score	2.0 ± 0.8	2.0 ± 0.8	2.0 ± 0.8	0.6043
SOFA-Cardiovascular score	1.5 ± 1.0	1.7 ± 1.0	1.5 ± 1.0	0.1284
SOFA-Central Nervous System score	2.0 ± 1.1	2.2 ± 1.0	1.9 ± 1.1	0.0094
SOFA-Renal score	0.8 ± 1.2	0.7 ± 1.1	0.8 ± 1.2	0.6669
SOFA-Coagulation score	0.4 ± 0.6	0.4 ± 0.7	0.3 ± 0.6	0.5521
SOFA-Hepatic score	0.4 ± 0.7	0.4 ± 0.7	0.4 ± 0.7	0.9780
Organ support				
Ventilation days/observation days (%)	66 ± 32	71 ± 32	65 ± 32	0.0280
Vasopressor days/observation days (%)	34 ± 30	37 ± 33	33 ± 29	0.2919
Dialysis days/observation days (%)	9 ± 23	9 ± 23	10 ± 22	0.8287
Inflammatory values				
Leucocytes (1000/µL)	13 ± 5	13 ± 5	13 ± 5	0.1026
CRP (mg/L)	152 ± 87	137 ± 81	156 ± 88	0.1356
Procalcitonin (ng/dL)	4.0 ± 9.3	3.5 ± 10.1	4.1 ± 9.1	0.0655
Kidney values				
Urine output (mL/day)	2978 ± 1337	2973 ± 1388	2979 ± 1324	0.9387
Urine output (mL/kg/day)	1.6 ± 0.8	1.6 ± 0.8	1.6 ± 0.78	0.9051
Creatinine (mg/dL)	1.2 ± 0.9	1.2 ± 0.9	1.3 ± 1.0	0.4218
Liver values				
AST (GOT) (IU/L)	169 ± 598	127 ± 238	182 ± 672	0.7476
ALT (GPT) (IU/L)	94 ± 188	101 ± 193	92 ± 187	0.2135
Bilirubin (mg/dL)	1.2 ± 2.1	1.2 ± 1.8	1.2 ± 2.1	0.5972

CRP, C-reactive protein; AST, aspartate transaminase; GOT, glutamic oxaloacetic transaminase; ALT, alanine transaminase; GPT, glutamate-pyruvate transaminase.

**Table 7:** Disease severity with regard to the CTLA-4 haplotype H1: TAA.

Variable	All (*n* = 644)	H1: TAA		*p*-Value
		Positive (*n* = 447)	Negative (*n* = 197)	
SOFA	7.0 ± 3.5	7.0 ± 3.6	7.1 ± 3.5	0.5088
SOFA-Respiratory score	2.0 ± 0.8	2.0 ± 0.8	1.9 ± 0.8	0.9796
SOFA-Cardiovascular score	1.5 ± 1.0	1.5 ± 1.0	1.5 ± 1.0	0.7476
SOFA-Central Nervous System score	2.0 ± 1.1	2.0 ± 1.1	1.9 ± 1.1	0.2731
SOFA-Renal score	0.8 ± 1.2	0.7 ± 1.1	0.9 ± 1.3	0.1230
SOFA-Coagulation score	0.4 ± 0.6	0.4 ± 0.6	0.3 ± 0.6	0.6741
SOFA-Hepatic score	0.4 ± 0.7	0.4 ± 0.7	0.4 ± 0.8	0.4264
Organ support				
Ventilation days/observation days (%)	66 ± 32	67 ± 32	65 ± 32	0.3607
Vasopressor days/observation days (%)	34 ± 30	33 ± 30	35 ± 30	0.6474
Dialysis days/observation days (%)	9 ± 23	9 ± 22	11 ± 23	0.1246
Inflammatory values				
Leucocytes (1000/µL)	13 ± 5	13 ± 5	13 ± 4	0.4087
CRP (mg/L)	152 ± 87	147 ± 81	162 ± 99	0.4562
Procalcitonin (ng/dL)	4.0 ± 9.3	3.5 ± 9.1	5.1 ± 9.7	0.0002
Kidney values				
Urine output (mL/day)	2978 ± 1337	3060 ± 1370	2792 ± 1242	0.0229
Urine output (mL/kg/day)	1.6 ± 0.8	1.6 ± 0.8	1.5 ± 0.8	0.0529
Creatinine (mg/dL)	1.2 ± 0.9	1.2 ± 0.9	1.4 ± 1.1	0.0902
Liver values				
AST (GOT) (IU/L)	169 ± 598	184 ± 687	131 ± 272	0.9910
ALT (GPT) (IU/L)	94 ± 188	99 ± 206	81 ± 142	0.1307
Bilirubin (mg/dL)	1.2 ± 2.1	1.2 ± 1.8	1.3 ± 2.5	0.9584
